# The Rise and Refinement of Breast Thread Lifting: A Contemporary Review

**DOI:** 10.3390/jcm14113863

**Published:** 2025-05-30

**Authors:** Razvan George Bogdan, Alina Helgiu, Vlad Adam Bloanca, Cristian Ichim, Samuel Bogdan Todor, Mihai Iliescu-Glaja, Horatiu-Paul Domnariu, Elisa Leonte, Zorin Petrisor Crainiceanu, Paula Anderco

**Affiliations:** 1 Department of Plastic Surgery, “Victor Babes” University of Medicine and Pharmacy Timisoara, 300041 Timisoara, Romania; razvan.bogdan@umft.ro (R.G.B.); mihai.iliescu.glaja@umft.ro (M.I.-G.); elisa.leonte@umft.ro (E.L.); crainiceanu.zorin@umft.ro (Z.P.C.); 2 County Clinical Emergency Hospital Pius Branzeu Timisoara, 300723 Timisoara, Romania; 3 Faculty of Medicine, “Lucian Blaga” University of Sibiu, 550169 Sibiu, Romania; cristian.ichim@ulbsibiu.ro (C.I.); samuelbogdant@gmail.com (S.B.T.); horatiupaul.domnariu@ulbsibiu.ro (H.-P.D.); paula.anderco@ulbsibiu.ro (P.A.); 4 County Clinical Emergency Hospital of Sibiu, 550245 Sibiu, Romania

**Keywords:** breast ptosis, thread lift, PDO threads, ultrasonography, minimally invasive breast surgery

## Abstract

Breast thread lifting is a minimally invasive technique for correcting mild to moderate ptosis, offering aesthetic enhancement with reduced morbidity compared to traditional mastopexy. This review examines the anatomical underpinnings, clinical indications, technical nuances and limitations of breast thread lifting. The breast’s fascial architecture, particularly the role of Cooper’s ligaments and the retromammary space, critically influences thread trajectory and vector planning. Classification systems assist in proper patient selection, highlighting the suitability of thread lifts for Grades I–II ptosis with minimal skin excess. Advances in ultrasonography have improved preoperative planning, thread placement accuracy and postoperative monitoring. Various thread types, including PDO, PLLA, PCL and Silhouette Soft, offer different lifting capacities and collagen-stimulatory properties, necessitating tailored material selection. Although thread lifts offer immediate improvements, their transient nature necessitates careful patient counseling to manage expectations regarding durability and potential maintenance sessions. Innovative techniques, including clavicular anchoring and multi-level subdermal scaffolding, have expanded the procedural repertoire. Despite certain limitations, breast thread lifting remains a valuable tool within the aesthetic surgeon’s armamentarium, particularly for patients seeking minimally invasive options with shortened recovery periods and favorable psychosocial outcomes. Future developments are expected to further enhance safety, reproducibility and long-term results.

## 1. Introduction

The field of aesthetic surgery has witnessed a continuous evolution of techniques aimed at addressing the multifaceted concerns of breast aesthetics, ranging from augmentation and reduction to mastopexy and oncoplastic reconstruction. Breast ptosis, defined as the descent of the breast gland over the thoracic wall, is a prevalent aesthetic and functional concern frequently addressed in plastic surgery. The condition arises due to multiple intrinsic and extrinsic factors, including age, gravity, pregnancy, significant weight loss, smoking, higher body mass index and larger bra cup size [1]. These factors contribute to the weakening of structural supports such as Cooper’s ligaments and the fascial system, leading to progressive tissue descent and loss of upper pole fullness [2].

Traditional surgical correction of ptosis via mastopexy, while effective, involves extensive tissue manipulation, longer recovery time and visible scarring [3]. In contrast, the breast thread lift has emerged as a minimally invasive alternative for treating mild to moderate ptosis, particularly for patients desiring subtle repositioning with minimal downtime or as an adjunct in postoperative maintenance [4].

Thread lifting has long been employed in facial rejuvenation, but its application in the breast area remains comparatively underexplored [4]. Notably, Dr. Roger Khouri introduced innovative techniques for breast reshaping, utilizing threads in conjunction with external expansion methods [5,6]. Khouri’s early work on the combination of external tissue expansion with thread-based and fat grafting techniques laid the foundation for modern, less invasive approaches to breast reshaping [5]. His contributions have significantly influenced the evolution of minimally invasive breast procedures.

The technique involves inserting barbed threads, either absorbable or non-absorbable, into the subcutaneous layer, where they provide mechanical lift and stimulate neocollagenesis. When properly placed, threads can restore the breast contour, improve symmetry and reposition the nipple–areolar complex without significant incisions [3]. Modern approaches to thread lifting benefit from an evolving understanding of breast fascial anatomy. The superficial and deep layers of fascia, the retromammary space and the anchoring role of Cooper’s ligaments all play critical roles in guiding thread trajectory and vector orientation [7].

In this review, we examine the anatomical, technical and clinical aspects of breast thread lifting, highlighting recent advancements, procedural nuances and the expanding role of imaging modalities. Through a comprehensive synthesis of the current literature, we aim to delineate both the promise and limitations of this innovative technique in aesthetic breast surgery.

## 2. Anatomical Breast Structure

The anatomical architecture of the breast is underpinned by the superficial fascial system, which provides essential structural support [8]. The superficial layer of this fascia envelops the breast parenchyma and resides in close proximity to the dermis, often rendering it indistinct from the overlying skin [9]. Conversely, the deep layer of the superficial fascia is more discernible and is situated along the posterior aspect of the breast. Interposed between this deep layer and the superficial layer of the deep (pectoral) fascia is the retromammary space, a loose areolar plane that facilitates the natural mobility of the breast over the thoracic wall [10].

The pectoral fascia, a component of the deep fascial system, overlays key musculature of the chest wall, including the pectoralis major, upper rectus abdominis, medial serratus anterior and the external oblique muscles in the lower central breast region [11]. Notably, the thickness of this fascial layer varies, being markedly thinner over the muscular segments of the pectoralis major and serratus anterior [12]. Extending from the deep muscular fascia into the breast parenchyma are Cooper’s ligaments, fibrous connective tissue structures that anchor to the dermis of the overlying skin [13]. These suspensory ligaments form a supportive network that maintains breast shape and position [7]. The relatively loose connections between the deep muscle fascia and the deep layer of the superficial fascia permit natural breast movement.

**Figure 1:**
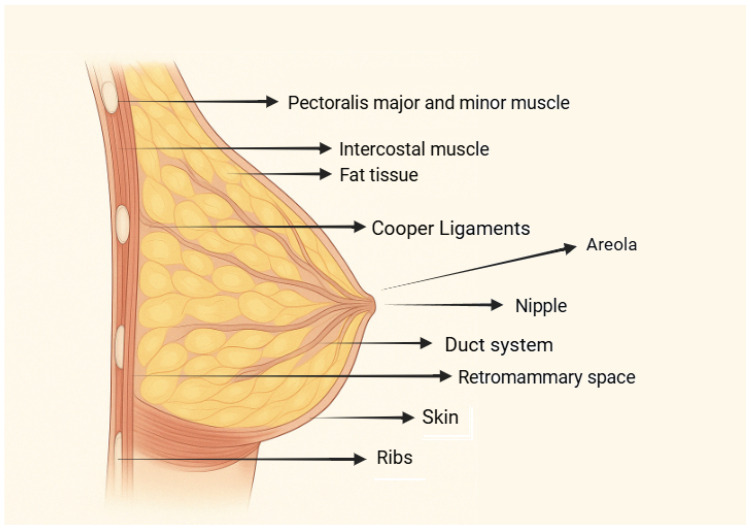
Breast anatomy.

The breast’s glandular tissue, responsible for milk production, is organized into lobules connected by ducts that lead to the nipple (Figure 1) [14]. This tissue is surrounded by subcutaneous fat, which shapes the breast and varies with age, hormones and body composition [14]. However, factors such as significant weight fluctuations, pregnancy and aging can lead to the attenuation and elongation of these attachments, resulting in breast ptosis and increased mobility [15]. This presents a considerable challenge in mastopexy procedures, particularly in patients who have experienced rapid weight loss exceeding 20 kg. In such cases, the diminished anchoring allows for extensive breast movement across the chest, often involving adjacent skin regions like the hypochondriac and epigastric areas. Consequently, meticulous preoperative planning and precise surgical marking become imperative to achieve optimal breast contour and repositioning [7].

## 3. Scientific Classification of Breast Ptosis

Breast ptosis, defined as the descent of the breast parenchyma and nipple–areola complex (NAC) in relation to the inframammary fold (IMF), is classified anatomically into distinct grades based on the relative position of the NAC. Regnault’s system remains a widely adopted clinical framework due to its simplicity, reproducibility and relevance in both aesthetic assessment and surgical planning [16]. Table 1 outlines the major ptosis grades along with their defining characteristics and clinical implications [16].

## 4. Challenges in Surgical Techniques

Surgical dissection in the upper pole of the breast presents notable technical challenges due to the dense anatomical integration and overlap of fascial structures in this region [9,17]. Specifically, the convergence of the pectoralis fascia, the deep layer of the superficial fascia and the superficial fascia of the breast near the second intercostal space results in a structurally complex and tightly bound region [12]. These fascial planes provide critical support and maintain breast position, extending superiorly toward the clavicle [18]. As such, procedures involving implant placement or reconstructive intervention demand meticulous dissection in this zone to prevent excessive mechanical tension and preserve the native anatomical integrity of the breast [7].

Although thread lifting is frequently described as “minimally invasive”, the procedure involves subcutaneous tissue manipulation, foreign material implantation and localized inflammatory reactions. These aspects contribute to its therapeutic effect but also introduce risks such as asymmetry, dimpling, thread migration, granuloma formation and localized infections [19,20]. Reported complication rates, though generally low, are not negligible and vary significantly with operator technique and experience. Therefore, the term “minimally invasive” should be interpreted cautiously and within the context of procedural variability.

Currently, one of the most pressing technical challenges is the lack of standardization in breast thread lifting. Variability in practitioner technique, insertion patterns and vector orientation contributes to inconsistent outcomes. The development of unified procedural guidelines remains essential to improve reproducibility and ensure patient safety [21].

The descent of the breast, including the nipple–areola complex, primarily affects the lower two-thirds of the vertical breast meridian and is attributed to cumulative factors such as aging, gravitational pull, increased breast volume and decreased tissue elasticity [8,22]. These factors compromise the structural support provided by suspensory ligaments and fascial layers, facilitating progressive ptosis and shape distortion [8]. As the breast envelope loses resilience, anatomical elements such as the breast mound and central pedicle adapt to gravitational forces, often exacerbating the degree of ptotic change [7]. While thread lifting may offer temporary improvement by repositioning superficial tissues, it does not modify the underlying degenerative mechanisms responsible for ptosis recurrence [23,24]. Continued action of gravitational and hormonal influences, along with ligamentous laxity, frequently results in the reappearance of sagging over time [1].

Ultrasound-based imaging has emerged as a valuable tool in addressing both anatomical complexity and technical variability [25]. High-resolution imaging allows for enhanced visualization of soft tissue architecture and has become instrumental in preoperative planning and postoperative assessment [26]. These capabilities may define future procedural benchmarks by improving both accuracy and long-term outcomes [27,28].

Since its introduction by Sulamanidze in the 1990s, thread lifting has evolved into a versatile modality featuring numerous design variants, including contour threads, ptosis-specific devices, Isse Endo Progressive Facelift Sutures and Silhouette Sutures [29]. These innovations have gained widespread acceptance among aesthetic practitioners [30].

The clinical effectiveness of thread lifting is governed by a complex interplay of variables, notably the diameter, tensile strength and material composition of the threads [20]. The design elements of barbs or cogs, including their size, orientation and distribution, are critical to achieving sufficient anchoring and lifting capacity [20,31]. Furthermore, insertion depth must be adapted to the mechanical properties of the underlying tissue to optimize support and ensure durable outcomes. Achieving a natural and symmetrical aesthetic result also requires careful planning of the lifting vectors and anchoring points [32].

Thread longevity, biocompatibility and the risk of adverse tissue responses influence the choice between absorbable and non-absorbable materials [33]. While non-absorbable threads offer extended support, they may be associated with long-term complications such as chronic inflammation or foreign body reactions [23]. In contrast, absorbable threads undergo gradual resorption and are generally better tolerated. Thicker threads with larger surface areas have demonstrated greater fibroblast and stem cell stimulation, contributing to enhanced neocollagenesis and lifting effects [33]. Among absorbable materials, poly-L-lactide-co-ε-caprolactone threads have shown promise in producing three-dimensional tissue repositioning in cases of mild to moderate breast ptosis [34,35]. Clinical trials report favorable outcomes, with high satisfaction rates among both surgeons and patients [20,36]. Adverse effects, when present, are typically minor and self-limiting, further supporting the safety and efficacy of this technique [37].

## 5. Indications for Surgical Procedures

The breast thread lift technique is primarily indicated for patients presenting with mild to moderate breast ptosis (Grades I–II according to standard classification systems), accompanied by a small to moderately sized breast volume (relative size 1–2.5) and minimal to moderate excess skin [38]. Candidates typically also exhibit early involutional changes in the breast parenchyma without significant loss of structural support [39]. In such cases, thread lifting offers a minimally invasive alternative for contour correction, nipple repositioning and volume redistribution without the morbidity associated with traditional mastopexy [40].

Breast thread lift procedures have been employed both as primary interventions for the correction of initial ptosis and as secondary treatments for the management of recurrent breast ptosis following previous surgical procedures [41]. Moreover, they can be utilized to address aesthetic concerns related to residual scarring postoperatively by enhancing tissue tension and contour [42].

In clinical practice, thread lifting is often combined with other techniques to optimize outcomes [23]. Commonly, combinations between skin retraction-based approaches and skin excision methods are employed depending on the degree of tissue laxity [20]. Natural skin contraction is harnessed in cases with good dermal elasticity, whereas direct excision is necessary when the dermal support has been irreversibly compromised due to progressive stretching of the parenchymal, adipose and superficial fascial structures over time [43,44]. This principle underlies not only traditional skin resection techniques but also contemporary endoscopic aesthetic procedures and hypodermic bodice techniques, which aim to maximize the skin’s intrinsic contractile capacity to achieve the desired aesthetic contour.

Furthermore, breast thread lift procedures can be effectively combined with autologous fat grafting. This adjunctive strategy allows for simultaneous enhancement of breast projection, restoration of symmetry and refinement of upper pole fullness, offering a comprehensive approach to minimally invasive breast rejuvenation [40].

To better contextualize the role of breast thread lifting compared to traditional mastopexy, Table 2 summarizes key differences between the two approaches, highlighting aspects related to invasiveness, recovery, risk profiles and durability of outcomes [45,46,47,48,49,50,51,52].

## 6. Ultrasonography Evaluation

Ultrasonography is a reliable and non-invasive imaging modality increasingly used in aesthetic procedures. It offers over 98% precision in evaluating soft tissue layers, making it superior to traditional palpation or visual inspection methods for assessing subcutaneous fat and structural integrity [53,54,55]. B-mode ultrasonography with an 8 MHz linear array transducer enables real-time, high-resolution imaging of breast tissue. This facilitates preoperative planning, especially in mapping fat thickness and identifying ideal thread trajectories [56,57].

In thread lifting, ultrasound guidance improves thread placement accuracy, reduces technical complications such as misdirection or asymmetry and supports postoperative monitoring [58,59,60]. Though long-term ultrasound-based follow-up is still limited, its utility in minimizing risks and enhancing precision is widely recognized [61,62]. Ongoing research into thread materials and insertion techniques increasingly integrates ultrasonographic feedback, suggesting its value in refining procedural safety and outcomes [23,33].

## 7. Thread Lifting Technique

### 7.1. Preoperative Assessment

A thorough pre-procedural evaluation is essential to ensure the safety and appropriateness of breast thread lift interventions. Clinical breast examination should be systematically performed for all candidates in order to exclude any preexisting breast pathology or structural anomalies [63,64]. The identification of malignancies, benign lesions or abnormal findings constitutes a contraindication to the procedure, as minimally invasive techniques like thread lifting are not therapeutic in nature and may delay necessary treatment for underlying conditions [63].

Following clinical clearance, the procedural field must be prepared aseptically to minimize infection risk [65,66]. Local anesthesia is typically administered approximately one hour before intervention, targeting the anticipated entry points of the threads to optimize patient comfort and procedural tolerance [2,67,68].

Despite the popularity of barbed suture techniques in aesthetic medicine, existing data suggest that while initial improvements in breast ptosis can be visually and subjectively significant, these effects tend to decline within 6–12 months, with most outcomes reverting toward baseline after one year [23,69]. This observation underscores the temporary nature of mechanical lift induced by threads, especially in dynamic anatomical regions such as the breast [70].

In parallel, a growing body of evidence highlights the psychosocial impact of aesthetic procedures, including thread lifts, across diverse patient populations [71,72]. These findings emphasize the need to incorporate embodiment theory and psychosocial context into pre-procedural counseling and expectations management [73]. While thread lifting may offer psychosocial benefits, its use must be restricted to anatomically and clinically appropriate cases.

### 7.2. Surgical Methods

The development of minimally invasive techniques for breast rejuvenation has led to the evolution of breast thread lift procedures, which offer an alternative to conventional surgical mastopexy. Several innovative methods of thread insertion have been employed to optimize breast contour, lift and firmness, depending on the specific anatomical requirements of each patient.

A combination of crosshatch threading, circular insertion and overlapping linear vectors is often utilized to achieve comprehensive structural support [23]. The crosshatch technique targets the upper pole and the area inferior to the nipple–areola complex, providing additional fullness and reinforcement in these regions [20]. Circular insertion in the upper pole enhances firmness and promotes superior breast shape through the creation of a scaffold-like network [17,74]. Furthermore, linear threading along vectors angled against gravity improves lift and longevity of the aesthetic result by mechanically counteracting downward forces [75]. Following the procedure, patients are typically advised to wear a supportive brace for two weeks to stabilize the tissues during the initial healing phase. Minor bruising is a recognized, self-limiting postoperative effect [2]. While thread lifting is generally considered less invasive than open surgical mastopexy, it is not without risks. Complications such as thread migration, extrusion, dimpling, asymmetry, hematoma, local infections and foreign body granulomas have been reported, with some requiring revision procedures [20,36].

The use of polydioxanone (PDO) threads has demonstrated immediate clinical improvements, with outcomes continuing to enhance over the first several weeks post-procedure [31]. Clinical evidence indicates that these results can persist for up to two years (18–20). Unlike traditional surgical techniques, thread lifts offer significant advantages, including shorter recovery times, lower complication rates and high patient satisfaction, contributing to their increasing popularity in aesthetic practice.

A unique contribution of thread lifting, particularly in breast rejuvenation, is the concept of scaffolding, a technique not previously described in classical mastopexy approaches [74,76]. By inserting threads at angles that oppose gravitational forces and by creating intersecting thread patterns, a collagenous matrix is stimulated, enhancing tissue support [20]. This method provides superior lifting capacity, particularly for patients with larger breast volumes. The definition and projection of the upper pole are further secured by circumferential insertion techniques [77]. Typically, a follow-up session is recommended after 12 to 15 months to maintain optimal results [2].

From a histological standpoint, PDO threads have been shown to stimulate fibroblast proliferation, collagen deposition and neoangiogenesis, contributing to enhanced skin texture and firmness [78]. Similar to other minimally invasive aesthetic interventions, such as botulinum toxin treatments, breast thread lifts require a high degree of procedural expertise, including precise patient selection, vector planning, accurate thread insertion into the Cooper’s ligaments and periodic maintenance sessions [62].

Breast thread lift has been increasingly recognized as an effective technique not only for correcting mild to moderate ptosis (Grades I–II) but also for addressing breast asymmetry without the need for excisional surgery or general anesthesia [79]. Importantly, PDO sutures are well-established in various surgical fields including orthopedic, ocular, gastrointestinal and cardiovascular surgeries, with a proven high tensile strength and biodegradation profile over six months, without associated carcinogenic risks [80]. This safety profile further supports their applicability in breast tissue repositioning.

Given that breast appearance and shape are critical to a woman’s self-esteem and overall psychological well-being, interventions that restore breast contour while minimizing surgical risks are of substantial clinical interest. While traditional procedures such as mastopexy and augmentation remain gold standards for significant ptosis correction, the breast thread lift offers a minimally invasive, low-risk alternative for patients seeking rejuvenation with minimal downtime [3].

### 7.3. Minimally Invasive Approach

Minimally invasive breast thread lift procedures have gained popularity among patients seeking to avoid the higher risks and prolonged recovery times associated with traditional surgical interventions [81]. Conventional breast augmentation techniques, particularly those involving silicone implants, are often complicated by capsular contracture and potential bacterial contamination during implant insertion [82]. Similarly, surgical innovations such as abdominoplasty–incision augmentation aimed to balance aesthetic improvements with functional recovery, addressing body changes related to pregnancy and weight fluctuations [83].

The breast thread lift is performed through small incisions, whereby specialized absorbable or non-absorbable threads are inserted using fine needles or cannulas. These threads are carefully positioned to lift and support the breast tissue, creating a natural and rejuvenated contour [4]. Compared to open surgical mastopexy, thread lifting offers significant advantages: reduced scarring, shorter recovery and minimized procedural morbidity.

Driven by evolving aesthetic trends, increasing patient demands and continuous technological innovation, minimally invasive techniques have emerged as viable alternatives to conventional surgical lifts [84]. Although their results may be slightly less dramatic compared to maximally invasive procedures such as full surgical mastopexy combined with liposuction, the trade-off in favor of reduced risks and faster return to daily activities is considerable [81]. The procedure is carried out under strict sterile conditions, typically under local anesthesia and may be supplemented with mild sedation depending on patient anxiety and procedural complexity.

Traditional surgical approaches to breast ptosis have involved numerous techniques, such as glandular release, parenchymal resections and various tissue redistribution methods [85,86]. Despite initial satisfactory results, many of these techniques have failed to provide durable correction, often due to inadequate support of the lifted tissue and progressive laxity of the suspensory skin envelope over time [87].

In contrast, structurally stable methods such as central pedicle reduction mammoplasty, introduced by Balch and refined by Hester, emphasize the preservation of the nipple–areola complex’s vascular and neural supply [88,89,90,91]. Würinger’s anatomical studies further reinforced the importance of protecting key neurovascular structures to ensure long-term viability [91]. Although more invasive, the central pedicle technique remains a benchmark due to its superior ability to maintain breast projection, shape and functionality [92,93,94].

Principles derived from these classic surgical methods, particularly regarding neurovascular preservation and anchorage stability, have indirectly informed the evolution of thread lift strategies. Techniques such as cellular endoprosthesis formation and clavicular fixation of the breast mound illustrate ongoing efforts to enhance structural support and long-term outcomes in minimally invasive breast rejuvenation [95,96].

## 8. Innovative Techniques

### 8.1. Thread Stabilization Technique

**Figure 2:**
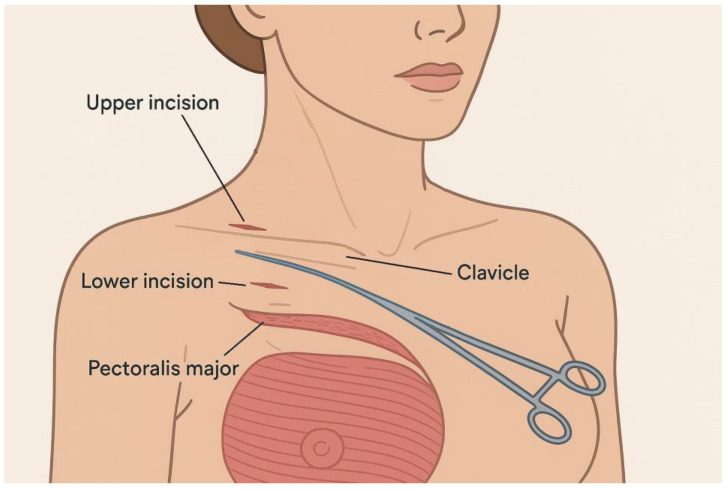
Thread Stabilization Technique.

A refined thread-based breast suspension method, initially documented in 2015, is typically conducted under general anesthesia [87]. The patient is positioned in a semi-reclined posture, facilitating anatomical access and gravitational balance. Two micro-incisions, each measuring approximately 2–3 mm, are strategically placed along the midclavicular axis (Figure 2):

- The inferior incision is situated at the level of the second intercostal space, adjacent to the fascia of the pectoralis major muscle.
- The superior incision lies just above the clavicle, directly over the periosteal sheath.

Using a fine mosquito clamp, the surgeon gently dissects the soft tissue planes, ensuring minimal trauma. To enhance tissue separation and limit bleeding, hydrodissection is performed with 20 mL of saline solution mixed with epinephrine, infiltrated around the clavicle.

A blunt-ended Aptos Needle (DRN 60) preloaded with polypropylene suture material is introduced through the lower incision. It is then advanced in a subdermal tunnel towards the upper incision, from which the needle emerges, pulling the thread along its path. A 10 cm suture tail is intentionally left at the lower access site to facilitate subsequent anchoring.

At the upper incision, the needle is carefully rotated and redirected to encircle the clavicle, adhering closely to its curvature to ensure firm fixation. The thread is anchored by creating a knot at the inferior site. The needle is then maneuvered in a postclavicular trajectory, gliding through the retrofascial plane of the pectoralis major muscle and finally redirected back to the initial entry point, exiting through the same incision. The procedure concludes with layered closure of the superior incision, using two interrupted 6/0 Prolene sutures to ensure optimal healing and minimal scarring [87].

### 8.2. Hypodermic Bodice Technique

The “hypodermic bodice” technique is a surgical approach to breast elevation that involves the insertion of a reticulated mesh implant within the subcutaneous tissue layer, under general anesthesia. The procedure begins with the patient placed in a semi-upright position to optimize anatomical orientation and facilitate tissue handling. Local infiltration of the hypodermis is performed using an isotonic saline solution containing epinephrine, enhancing the dissection planes and minimizing intraoperative bleeding.

**Figure 3:**
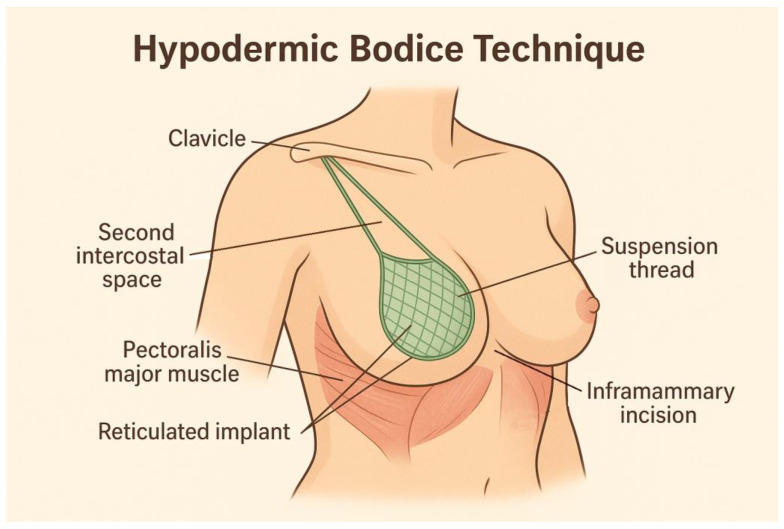
Hypodermic bodice technique.

Access to the subcutaneous plane is obtained via an inframammary or periareolar incision, with the incision length adapted to the chosen dissection method (Figure 3). When blunt dissection is performed without endoscopic guidance, the incision typically measures around 5 cm, whereas endoscopic techniques or wire scalpel use allow for smaller entry points, approximately 2 to 3 cm. Once access is gained, a cellulocutaneous flap is mobilized within the lower medial and lateral breast quadrants. A reticular mesh is inserted and spread evenly beneath the flap once hemostasis is achieved.

The mesh is anchored to the lower edge of the areola and to the inframammary fold using interrupted sutures. To achieve upper pole support and lift, three to four pairs of suspension threads fixed to the medial and lateral aspects of the implant are tunneled subcutaneously through the intercostal space toward the clavicle. These threads are drawn up, tensioned and tied securely to pre-established clavicular sutures. Two additional interrupted stitches are placed at the second intercostal level for further stabilization and the primary incisions, subareolar and inframammary, are closed meticulously in three layers using a continuous suturing technique to promote optimal healing and aesthetic outcome [87].

### 8.3. Multi-Level Subdermal Breast Elevation with Clavicular Thread Anchoring

This advanced breast lifting technique utilizes a geometrically structured multi-point suspension system based on subdermal threading, designed to elevate and reshape the breast in a natural and aesthetically harmonious manner. The procedure begins with the drawing of a vertical midclavicular reference line, extending from the nipple to the inframammary fold. Along this central axis, four anatomical landmarks are delineated: Point A (located at the second intercostal space), the superior and inferior margins of the areola and additional predefined points referred to as B, C and D.

To establish a three-dimensional mapping of the breast contour, arched lateral guide lines are drawn to connect each vertical point (A through D) to their corresponding inferior points (A1 through D1), generating a teardrop-shaped overlay that demarcates the zones of suture support.

At each superior point (A–D), bayonet-shaped incisions approximately 1–1.5 cm in depth are made, followed by blunt dissection using a fine mosquito clamp to gently widen the entry sites. Threading is initiated at Point A, where an Aptos 2/0 needle is introduced and guided subdermally along the previously marked arcs. The needle is periodically withdrawn and redirected to form looped subcutaneous threads, ultimately returning to the initial entry point.

This process is systematically repeated for points B through D, creating concentric subcutaneous thread layers that span between each point and its corresponding inferior counterpart (e.g., B–B1, C–C1, D–D1). These layers provide graduated support to the lower breast pole, enhancing both stability and lift. Tensioning and tying of the sutures are performed sequentially, starting at point A and continuing through all levels, culminating in the convergence of all threads at Point E, the designated clavicular fixation site.

To secure the suspension, threads from points A, B and C are redirected toward the clavicle using a blunt-tipped Aptos DRN 60 needle, where they are anchored to a pre-established clavicular fixation thread. This ensures a stable and symmetric elevation of the breast, restoring both volume and contour without requiring skin excision.

Critical technical considerations include ensuring subdermal thread placement to avoid dermal penetration, minimizing visible retractions at exit sites and maintaining symmetrical, horizontal anchoring to preserve the natural anatomical breast form [87].

## 9. Thread Materials

The selection of appropriate thread material is a critical determinant of clinical outcomes in breast thread lifting procedures. Different threads vary not only in terms of composition and biodegradation profiles, but also in mechanical support capacity and biostimulatory properties. Table 3 summarizes the key features of commonly used thread types, highlighting their duration of effect, clinical applications and tissue support strength [23,31,46,50,70,75,84,97,98,99,100,101,102]. This comparative overview assists practitioners in selecting the optimal material based on the specific anatomical needs and aesthetic goals of each patient.

## 10. Limitations and Considerations

Thread lifting procedures offer primarily short-term aesthetic improvements and presenting them as equivalent alternatives to long-term surgical correction may raise ethical concerns. Patients typically seek medical care for durable outcomes and when transient effects are communicated without appropriate context or framed as definitive solutions, this may compromise informed consent and erode trust. It is therefore imperative that clinicians emphasize the temporary nature of results, the possibility of recurrence and the need for repeat procedures when counseling candidates for thread-based interventions.

While breast thread lifting represents a valuable minimally invasive option for breast contour enhancement, it is not without important limitations. One of the primary considerations is that thread lifts offer predominantly short-term solutions, particularly suited for mild cases of breast ptosis. The efficacy of the procedure is significantly reduced in the presence of excessive skin redundancy, making proper patient selection critical to achieving satisfactory outcomes [90].

Histological studies on PDO threads have provided insights into the biological responses elicited by different thread configurations. Threads composed of multiple intertwined strands stimulate higher collagen production compared to single-strand threads, with collagen synthesis peaking approximately two weeks after implantation and continuing to rise through the three-month mark [103]. However, the type of material also plays a role: mono-PLA threads have demonstrated superior biostimulatory effects compared to mono-PDO threads, while spring-shaped PDO threads have been less effective [103]. Therefore, the careful selection of thread type, diameter, architecture and insertion technique is paramount to optimize both the mechanical lift and the regenerative response.

Comparative histological analyses have further revealed that multiple-strand threads produce larger and more distinct structural elements within the tissue. The implantation of PDO threads leads to an initial accumulation of fibroblasts and inflammatory cells, peaking around two weeks post-procedure. By twelve weeks, the inflammatory response diminishes substantially; however, a higher prevalence of multinucleated giant cells and histiocytes is noted particularly around quadruple-thread configurations [103]. This phenomenon suggests a more pronounced foreign body reaction, which may prolong tissue remodeling and influence long-term aesthetic outcomes.

Given these biological dynamics, clinicians must weigh the benefits of enhanced collagen stimulation against the potential for increased tissue reactivity when selecting thread configurations. Additionally, realistic patient counseling regarding the duration of results, the possibility of requiring maintenance procedures and the limitations related to skin excess remains essential for achieving high levels of patient satisfaction. The long-term effects of repeated breast thread lift sessions are unclear. Emerging concerns include potential fibrosis and altered tissue quality, which may affect outcomes of subsequent surgeries.

## 11. Conclusions

Breast thread lifting represents a promising, minimally invasive modality for the aesthetic rejuvenation of mild to moderate breast ptosis. Rooted in an intricate understanding of breast fascial anatomy, modern thread lifting techniques offer meaningful improvements in breast contour, symmetry and nipple–areolar positioning with reduced downtime and surgical morbidity. The integration of ultrasonography and evolving thread materials, such as PDO and PLLA, has enhanced procedural precision and extended the durability of results. Nevertheless, limitations persist, including the temporary nature of lift, variable tissue responses and reduced efficacy in cases with significant skin excess.

Careful patient selection, realistic counseling and meticulous technique are critical to optimizing outcomes and minimizing complications. While thread lifting cannot fully replace traditional surgical mastopexy in severe ptosis cases, it offers a valuable alternative for selected patients prioritizing minimally invasive approaches. Ongoing innovations in thread design, insertion methods and adjunctive therapies are likely to expand the indications and improve the predictability of this technique. Future studies focusing on long-term outcomes and standardized protocols will be essential to further consolidate the role of breast thread lifting within aesthetic breast surgery.

## Figures and Tables

**Table 1:** Classification of breast ptosis.

Grade	Nipple Position Relative to Inframammary Fold	Glandular Contour Characteristics	Clinical Notes
Grade I	At the level of or ≤1 cm below the IMF	Nipple is still above the lower contour of the breast mound	Mild ptosis, often aesthetic concern only
Grade II	>1 cm below the IMF but still above the lower contour of the breast	Nipple lies midway between the IMF and the inferior breast contour	Moderate ptosis, frequently an indication for surgical correction
Grade III	At or below the lowest point of the breast mound	Nipple points downward, typically at the lowest point of the gland	Severe ptosis, requires full surgical mastopexy
Pseudoptosis	At or above the IMF	Glandular tissue descends below IMF while nipple remains in normal position	Often post-lactational or post-weight loss, not true ptosis
Bottoming Out	Nipple remains elevated	Breast parenchyma has migrated below IMF	Often seen post-surgery (augmentation/reduction), may need revision

**Table 2:** Breast thread lift vs. traditional mastopexy.

Feature	Breast Thread Lift	Traditional Mastopexy
Level of Invasiveness	Minimally invasive (no skin excision)	Invasive (extensive skin and parenchymal excision)
Estimated Duration of Results	12–24 months (depending on material and technique)	10–15 years or longer
Recovery Time	1–2 weeks	4–6 weeks
Risks and Complications	Asymmetries, inflammatory reactions, thread displacement	Visible scarring, NAC necrosis, ptosis recurrence
Ideal Candidates	Mild to moderate ptosis (Grade I–II)	Moderate to severe ptosis (Grade II–III)
Anesthesia Requirement	Local anesthesia ± mild sedation	General anesthesia
Immediate Aesthetic Results	Subtle and natural improvement	Complete correction and structural reshaping
Estimated Cost	Moderate	High

**Table 3:** Characteristics of common thread types used in breast lifting procedures.

Thread Type	Composition	Duration of Effect	Clinical Features	Tissue Support Strength	Repeated Use Considerations
PDO	Biodegradable polymer	6–12 months	Quick collagen boost; best for early signs of skin laxity	Mild	Limited long-term data; repeated use may increase fibrosis risk
Poly-L-Lactic Acid (PLLA)	Biodegradable poly-L-lactic acid	12–18 months	Sustained collagen stimulation; suitable for midface volumization	Moderate	Reuse may affect tissue quality; long-term effects unconfirmed
Polycaprolactone (PCL)	Biodegradable polycaprolactone	18–24+ months	Deep structural reinforcement; extended lifting performance	Strong	Repeated use insufficiently studied; potential for delayed tissue changes
Silhouette Soft	Polylactic acid with molded cones	18–24 months	Dual effect: repositioning and collagen stimulation via cones	Moderate to strong	Possible fibrotic response with multiple sessions; caution recommended
Barbed Sutures	Permanent synthetic fiber	Long-term/permanent	Best for significant tissue drooping; durable mechanical hold	Very strong	Not advised for repeat sessions due to chronic inflammation risk
